# Osteonecrosis of the femoral heads in patients with systemic onset juvenile idiopathic arthritis: comparison of the results of radiological and morphological methods

**DOI:** 10.1186/1546-0096-9-S1-P134

**Published:** 2011-09-14

**Authors:** D Alexeev, I Nikishina, A Smirnov, L Semenova, L Bogjeva, S Makarov, S Radenska-Lopovok, D Ivanov

**Affiliations:** 1 Research Institute of Rheumatology of Russian Academy of Medical Sciences, Moscow, Russian Federation

## Background

The destruction of the hip joints with the development of osteonecrosis (ON) - a common complication and a leading cause of functional impairment in patients with systemic onset juvenile idiopathic arthritis (soJIA).

## Aim

A detailed study of the character of local disturbances and the structure of femoral heads (FH) in soJIA according to the comprehensive radiologic and morphological studies.

## Methods

Four patients with similar clinical and demographic characteristics (female, age 17-20 years, soJIA, disease duration >10 years, the duration of hip damage > 5 years), underwent total hip replacement (5 hip joints), because of ON of the FH. Changes in the FH were investigated using a specially designed protocol with the use of radiologic (conventional radiography (Ro), computed tomography (CT), magnetic resonance imaging (MRI)) and morphological techniques.

## Results

Ro-, CT revealed pronounced joint space narrowing, the picture of osteonecrosis with FH deformation, realignment of bone structure, erosions. Zones with significant restructuring of the bone (detected by CT) and enhanced heterogeneous MRI signal (T1-, T2- weighted) were characterized by diverse damage in cartilaginous and bone tissues at morphologic examination (fissures in cartilage; cysts, sclerosis, necrosis of trabecular bone).

**Figure 1:**
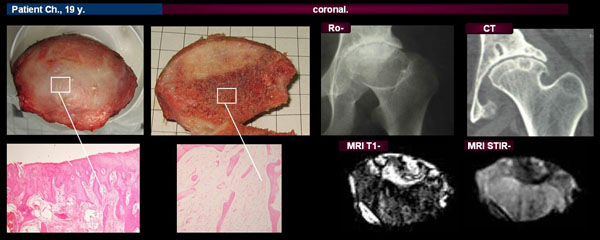


## Conclusion

The results show that in patients soJIA with destructive hip damage similar changes of FH, interpreted by the radiological methods as ON, are characterized by a variety of histological disorders. Further in-depth comprehensive study of bone structure, damaged by ON of the FH will promote understanding of the pathogenetic nature of this complication.

